# The dual roles of *Prevotella* in gastrointestinal tumorigenesis: from molecular mechanisms to therapeutic prospects

**DOI:** 10.3389/fonc.2026.1855577

**Published:** 2026-08-28

**Authors:** Zixuan Lu, Dong Tang

**Affiliations:** 1 The First School of Clinical Medicine, Faculty of Medicine, Yangzhou University, Yangzhou, China; 2 Department of General Surgery, Northern Jiangsu People’s Hospital Affiliated to Yangzhou University, Yangzhou, Jiangsu, China

**Keywords:** biomarkers, chronic inflammation, gastrointestinal tumors, gut microbiome, *Prevotella*, short-chain fatty acids (SCFAs), tumor immunity, tumor microenvironment

## Abstract

Tumor microbiology is a prominent area of research that is reshaping cancer treatment paradigms. While most microbiological studies have focused on specific bacterial species such as *Fusobacterium nucleatum*, the genus *Prevotella* has emerged in recent years as a key research focus due to its notable functional duality in gastrointestinal tumorigenesis. The role of this genus is not unidirectional; specific strains of *Prevotella* have been associated with either pro-tumorigenic or anti-tumorigenic effects depending on the context. On one hand, pro-tumorigenic strains, exemplified by *Prevotella intermedia*, have been shown to correlate with malignant progression through various mechanisms, though direct causal evidence in humans remains limited. On the other hand, certain *Prevotella* strains may exert protective effects primarily through the production of short-chain fatty acids (SCFAs), particularly butyrate, as suggested by preclinical and correlational human studies. This functional duality is fundamentally shaped by strain-level heterogeneity, dietary patterns, microbial community context, and anatomical localization. Despite the critical importance of this genus in the gastrointestinal tract, a comprehensive synthesis of its strain-specific and context-dependent roles remains lacking. This narrative review is based on literature searched in PubMed and Web of Science up to March 2026 using keywords including “Prevotella”, “gastrointestinal cancer”, “short-chain fatty acids”, and “gut microbiota”. Additional references were identified from reference lists. No formal bias assessment was performed. Therefore, this review summarizes the current understanding of the underlying mechanisms of its dual roles—emphasizing where evidence is associative versus causal—and explores emerging translational strategies that leverage this duality for cancer prevention and therapy.

## Graphical Abstract

**Figure:**
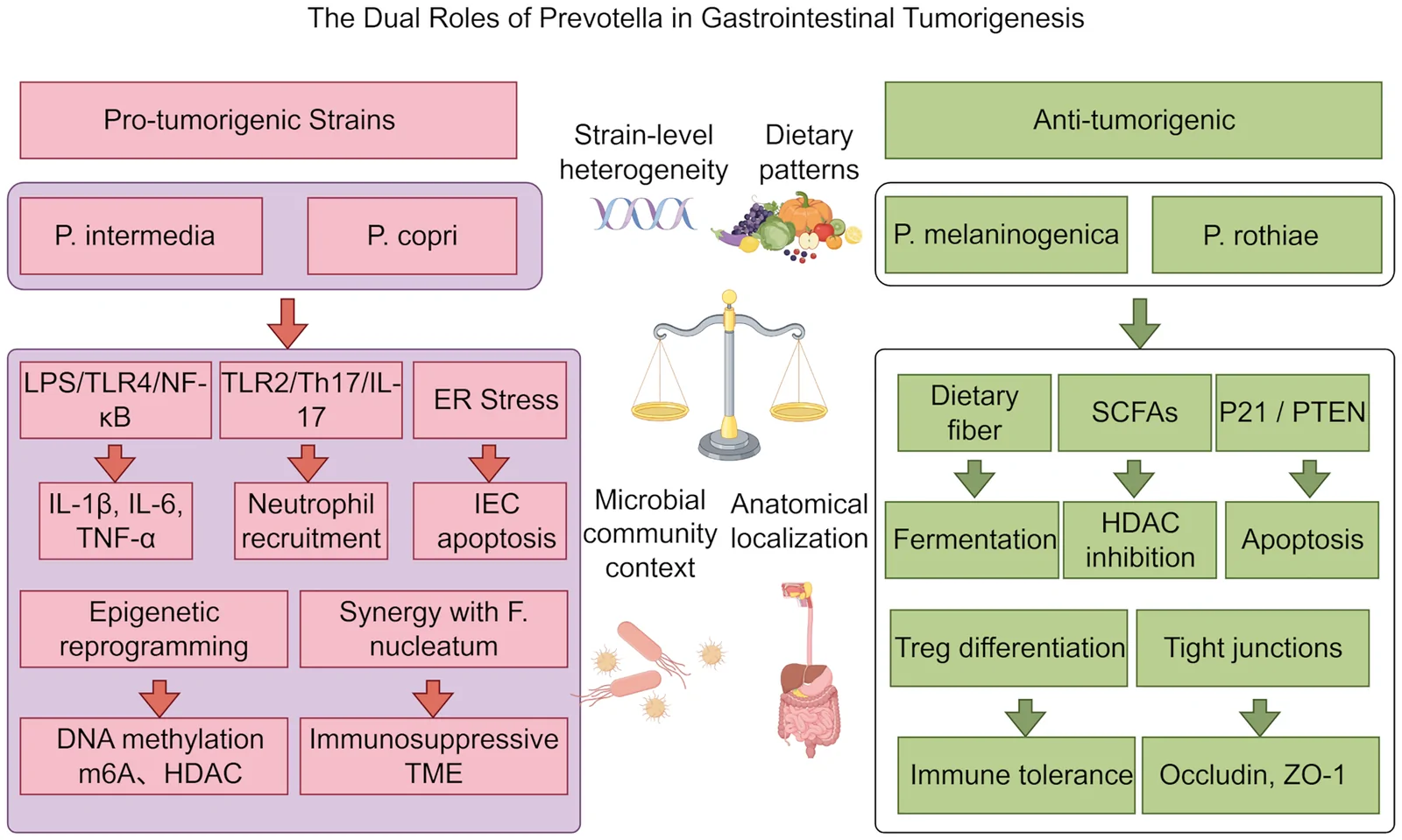
Balancing the dual roles of Prevotella in gastrointestinal tumorigenesis. This graphical abstract summarizes the dual roles of Prevotella in gastrointestinal tumor development. The left panel (red) illustrates pro-tumorigenic mechanisms driven by specific strains such as P. intermedia and P. copri, including: (1) activation of TLR4/NF-κB and TLR2/Th17/IL-17 inflammatory axes; (2) synergistic interactions with Fusobacterium nucleatum; (3) epigenetic reprogramming (DNA methylation, m6A modification, histone modification); and (4) endoplasmic reticulum stress-induced epithelial cell apoptosis. The right panel (green) illustrates anti-tumorigenic mechanisms mediated by SCFA-producing strains such as P. melaninogenica and P. rothiae, including: (1) fermentation of dietary fiber to produce butyrate; (2) histone deacetylase inhibition leading to p21/PTEN upregulation and cell cycle arrest; (3) promotion of regulatory T cell differentiation; and (4) enhancement of intestinal barrier function via tight junction proteins (occludin, ZO-1). The central scale indicates that the net outcome is determined by four key factors: strain-level heterogeneity, dietary patterns, microbial community context, and anatomical localization. SCFAs, short-chain fatty acids; HDAC, histone deacetylase; Tregs, regulatory T cells; ER, endoplasmic reticulum.

## 1 Introduction

Globally, gastrointestinal cancers account for 2.5 million new cases annually and represent a leading cause of cancer-related mortality, posing a serious threat to human health (1). Consequently, researchers are actively investigating early detection methods and effective treatment strategies for these malignancies, including emerging approaches such as immune checkpoint inhibitors (2) and cell-based therapies (3, 4). Despite the considerable efficacy of these treatments (5), a substantial proportion of patients exhibit poor responses or develop resistance to immunotherapy. In this context, the gut microbiota has emerged as a critical focus for the development of novel therapeutic strategies. As an integral component of the human digestive tract, the gut microbiota influences the initiation and metastasis of gastrointestinal tumors and has been established as an indispensable active participant in the tumor microenvironment (6, 7). Through mechanisms involving microbial metabolites, immune modulation, and the gut–brain axis, the gut microbiota profoundly affects host physiology and pathological states (8–10). Disruption of this symbiotic balance, termed dysbiosis, may drive tumorigenesis by inducing chronic inflammation, producing genotoxins, and mediating immune evasion (11).

Among the myriad microorganisms inhabiting the human body, the genus *Prevotella* has garnered increasing attention due to its recent identification as a colonizer of multiple critical sites (12), where certain species within this genus have been implicated in a range of disease processes. One well-studied example is *Prevotella intermedia*, a Gram-negative, black-pigmented anaerobic bacterium (13) characterized as a non-spore-forming, non-flagellated short rod with saccharolytic or moderately saccharolytic capabilities (14). Within the human microbiome, different *Prevotella* species exhibit high abundance across various anatomical sites and are thought to play pivotal roles in maintaining the balance between health and disease, though these roles vary by species and context (12). In humans, *Prevotella* species have been found to colonize multiple body sites, including the skin, oral cavity (15), vagina, and gastrointestinal tract (16). Notably, members of the genus *Prevotella* often dominate the gut microbiota in rural populations that adhere to pre-industrial “traditional” lifestyles and diets—referred to as “non-Westernized populations” (17).

However, unlike many pathogenic bacteria with relatively singular functions, the genus *Prevotella* as a whole exhibits a paradoxical role in oncology, primarily because different species and strains have distinct, even opposing, effects. A substantial body of research has linked specific *Prevotella* species (e.g., *P. intermedia*) to inflammatory diseases such as rheumatoid arthritis, suggesting their pro-tumorigenic potential (18). Conversely, other studies have indicated that certain *Prevotella* strains, owing to their potent capacity to ferment dietary fiber, produce short-chain fatty acids with anti-inflammatory and anti-cancer properties, thereby potentially assuming a protective role (19, 20). This article provides a comprehensive overview of recent advances in *Prevotella* research, exploring how this genus and its strain-specific metabolites influence the functions of the healthy digestive tract. It systematically delineates the functional differences among *Prevotella* species and strains colonizing distinct anatomical sites and, finally, delves into their implications for the treatment of gastrointestinal tumors.

## 2 Mechanisms of *Prevotella*-driven inflammation and carcinogenesis

The pro-tumorigenic effects of *Prevotella* are not mediated through a single pathway but are driven by a coordinated network involving chronic inflammation, microbial interactions, epigenetic regulation, and trans-organ translocation. However, it is important to note that most mechanistic evidence comes from studies on specific pro-tumorigenic species, such as *Prevotella intermedia*, or from animal models using defined strains. Certain *Prevotella* strains (primarily those associated with dysbiosis and inflammation) have been shown to induce chronic inflammation in the digestive tract through various inflammatory signaling pathways (13). Subsequently, a range of inflammatory factors, microbial metabolites, and bacterial toxins generated in the inflamed digestive tract create a tumor-permissive microenvironment, thereby facilitating tumor initiation.

### 2.1 Induction and persistence of chronic inflammation by *Prevotella* via specific inflammatory pathways

The induction and persistence of chronic inflammation serve as the central link in the pro-tumorigenic mechanism of certain *Prevotella* strains (e.g., pro-inflammatory species such as *P. intermedia; Prevotella copri*), directly connecting bacterial colonization to malignant transformation of host cells. This section integrates mechanisms such as the LPS/TLR4/NF-κB signaling pathway, the TLR2/Th17/IL-17 axis, endoplasmic reticulum stress, and microbial interactions (**Table 1**) to elucidate how these pro-tumorigenic *Prevotella* strains contribute to inflammation.

**Table 1:** Overview of the dual role of *Prevella* in cancer.

Type of effect	Key molecules/microbial taxa	Core mechanisms	Downstream effects	Clinical associations	References
Pro-tumorigenic (97)	LPS, TLR2/4, IL-1β, IL-6, IL-17, TNF-α	Activation of TLR4/NF-κB and TLR2/Th17/IL-17 axes, chronic inflammation	Pro-inflammatory cytokine-rich microenvironment, DNA damage,	Increased risk of gastric and colorectal cancers (strain-specific)	(98)
	F.nucleatum	Synergistic interaction with bacteria, amplifying inflammation	Immunosuppressive TME (MDSC recruitment), chemoresistance	Oral-GI axis involvement in esophageal and pancreatic cancer risk	(42)
	DNMTs, METTL3	Inflammatory milieu-induced epigenetic reprogramming	Tumor suppressor silencing, oncogene stabilization, PD-L1-mediated immune evasion	Advanced stage, 5-FU resistance, poor prognosis	(6, 99)
	Succinate, HIF-1α, CHOP	Metabolic perturbation-induced ER stress and unfolded protein response	Intestinal epithelial apoptosis, barrier disruption	Contribution to IBD pathogenesis and CRC risk	(33)
Antitumor	SCFAs, metabolites	Induction of tolerogenic trained immunity via epigenetic reprogramming of innate cells	Sustained anti-inflammatory tone limiting inflammation-driven carcinogenesis (100)	Broad protective potential against inflammation-associated cancers (101)	(20)

#### 2.1.1 Activation of the LPS/TLR4/NF-κB signaling pathway promotes inflammation

**Figure 1:**
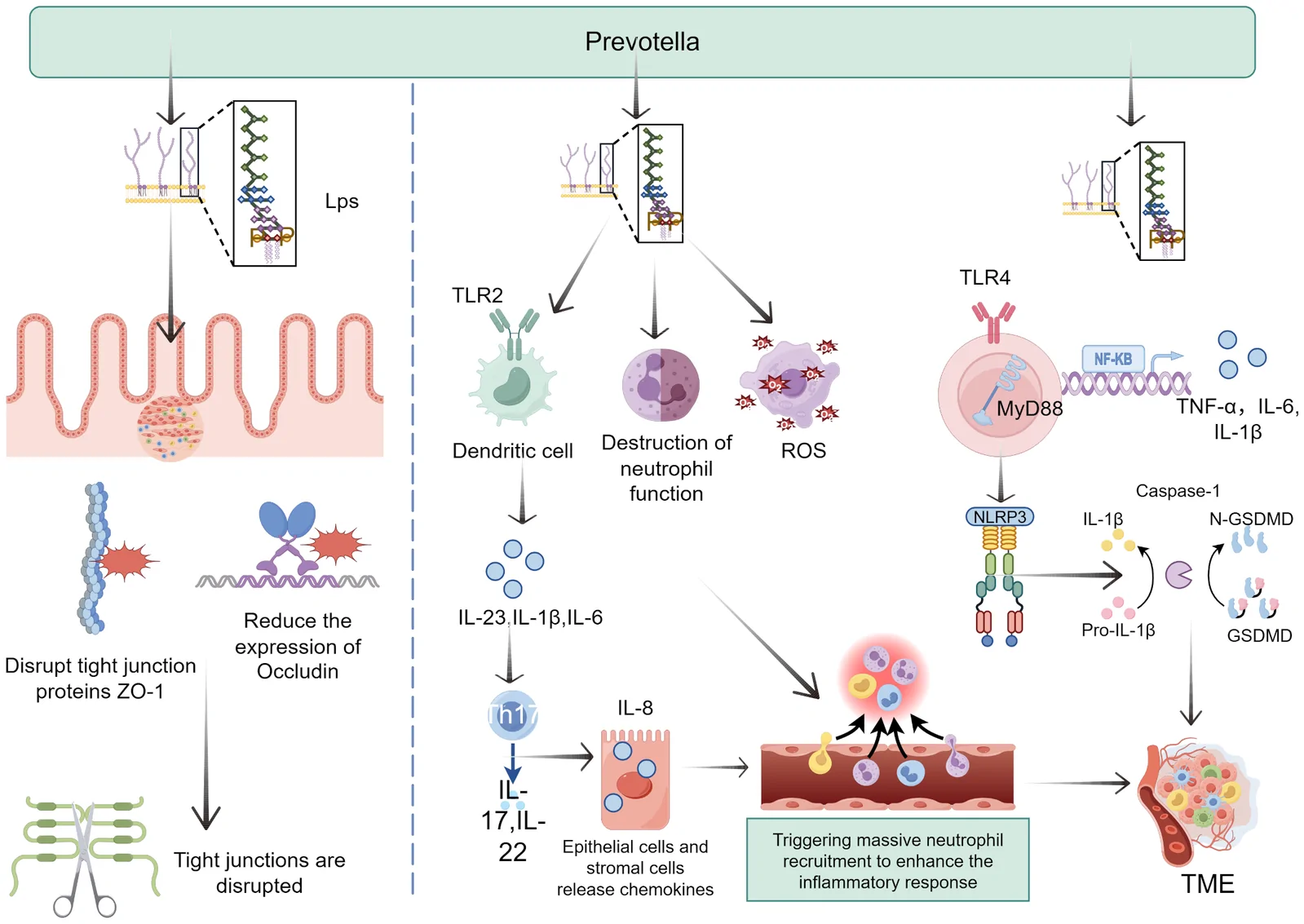
Mechanisms of chronic inflammation driven by *Prevotella* via the TLR4/NF-κB and TLR2/Th17/IL-17 signaling axes. The classical pathway by which *Prevotella* lipopolysaccharide induces inflammation via the TLR4/NF-κB pathway: barrier disruption leads to LPS translocation, which binds to the TLR4/MD-2 receptor and activates downstream NF-κB signaling, thereby promoting NLRP3 inflammasome assembly and the release of key pro-inflammatory factors such as IL-1β, IL-6, and TNF-α. Concurrently, the non-canonical pathway in which *Prevotella* activates dendritic cells via TLR2, drives Th17 cell differentiation and the production of IL-17/IL-22, and subsequently recruits neutrophils to amplify inflammatory responses. Through these multiple signaling cascades, *Prevotella* collectively shapes a chronic inflammatory microenvironment conducive to tumorigenesis.

Chronic inflammation serves as a critical bridge linking microbial infection to tumor initiation and progression (21). As Gram-negative anaerobic bacteria, certain *Prevotella* species can exert pro-inflammatory and potentially pro-tumorigenic effects through lipopolysaccharide (LPS) located in their cell wall. A well-characterized example is *Prevotella copri*, whose LPS (Pc-LPS) has been shown to drive such effects (22). Impairment of intestinal barrier function (e.g., reduced expression of tight junction proteins ZO-1 and occludin) facilitates the translocation of *P. intermedia*-derived LPS into the bloodstream. Elevated serum LPS levels further activate systemic inflammatory responses. Pc-LPS binds to TLR4 on vascular smooth muscle cells (VSMCs) and in aortic tissue. TLR4 is a key pattern recognition receptor (PRR) for LPS (23), and its expression is significantly upregulated following *P. intermedia* colonization. Upon TLR4 activation, the downstream NF-κB signaling pathway (22) is strongly activated, as evidenced by: 1) increased phosphorylation of NF-κB (p-NF-κB); and 2) nuclear translocation of NF-κB, which promotes the transcription of inflammatory genes. Activation of the NF-κB pathway further promotes the assembly and activation of the NLRP3 inflammasome (24). Following NLRP3 inflammasome activation, caspase-1 is activated, which subsequently cleaves pro-IL-1β and pro-IL-18 to generate their active forms. The activated NF-κB and NLRP3 pathways lead to the secretion and release of multiple pro-inflammatory cytokines, including TNF-α, IL-6, and IL-1β, which collectively amplify the inflammatory response (25, 26) and create a local chronic inflammatory environment that contributes to the development of gastrointestinal cancers (**Figure 1**). It is important to note, however, that most of these mechanistic insights are derived from studies on *P. intermedia* or other specific pro-inflammatory *Prevotella* species; whether all *Prevotella* species share this capability remains unclear (27).

#### 2.1.2 The *Prevotella*-mediated core inflammatory pathway: the TLR2/Th17/IL-17 Axis drives chronic inflammatory responses

A principal mechanism underlying the pathogenicity of pro-inflammatory *Prevotella* species(*P. intermedia*)at various mucosal sites lies in its potent activation of the Toll-like receptor 2 (TLR2)-driven T helper 17 (Th17) pathway (28). Unlike many Gram-negative pathogens that signal through TLR4 via hexa-acylated lipopolysaccharide (LPS), *P. intermedia* possesses penta-acylated LPS, rendering it a weak TLR4 agonist (29). Instead, its pathogen-associated molecular patterns are primarily recognized by TLR2 on antigen-presenting cells, particularly dendritic cells. This engagement initiates the production of key Th17-polarizing cytokines, including IL-23, IL-1β, and IL-6 (27, 30). This cytokine milieu may drives the differentiation of naïve T cells into Th17 cells, which subsequently amplify inflammatory responses through the secretion of IL-17 and IL-22. As a critical effector molecule, IL-17 induces the release of chemokines such as IL-8 (27) from epithelial and stromal cells, leading to substantial neutrophil recruitment. Furthermore, *P. intermedia* directly impairs neutrophil function by diminishing phagocytosis and reactive oxygen species production, thereby compromising bacterial clearance and contributing to the persistence of a chronic inflammatory state (27). This core pathway—*P. intermedia*→ TLR2 → dendritic cells → (IL-23/IL-1β/IL-6) → Th17 → IL-17 → neutrophil recruitment/dysfunction—underpins the pathological changes associated with *P. intermedia* in inflammatory bowel disease (**Figure 1**).

#### 2.1.3 *Prevotella* activates endoplasmic reticulum stress in intestinal epithelial cells, thereby initiating and sustaining inflammatory states

**Figure 2:**
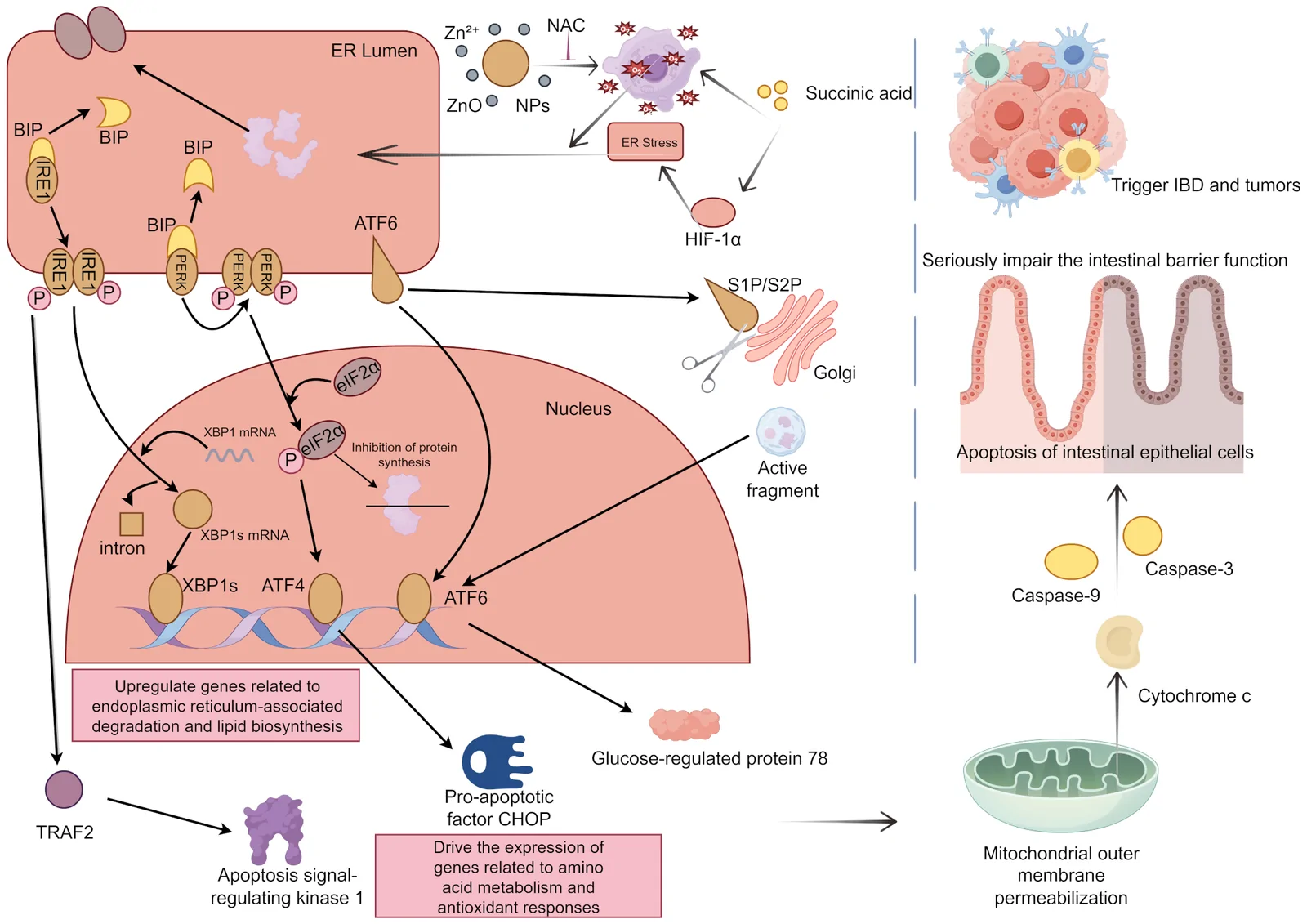
*Prevotella* induces endoplasmic reticulum stress and the unfolded protein response through metabolic reprogramming. *Prevotella* metabolizes dietary fiber, leading to succinate accumulation. Following uptake by intestinal epithelial cells, succinate inhibits prolyl hydroxylase, stabilizes HIF-1α, and induces a “pseudo-hypoxic” state that increases the protein synthesis burden, thereby triggering endoplasmic reticulum stress. IRE1α-XBP1s pathway: Endoribonuclease activity is activated, resulting in non-canonical splicing of *XBP1* mRNA. Spliced XBP1 acts as a potent transcription factor that upregulates genes involved in endoplasmic reticulum-associated degradation and lipid biosynthesis. However, under persistent stress, IRE1α also recruits TNF receptor-associated factor 2, activating the apoptosis signal-regulating kinase 1 and c-Jun N-terminal kinase pathway. PERK-ATF4-CHOP pathway: Protein kinase R-like endoplasmic reticulum kinase dimerizes and autophosphorylates, leading to phosphorylation of eukaryotic translation initiation factor 2α. This globally attenuates cap-dependent translation to reduce the nascent protein load, while selectively enabling the translation of activating transcription factor 4, which drives the expression of genes involved in amino acid metabolism and antioxidant responses. Prolonged ATF4 signaling upregulates the pro-apoptotic factor CHOP. ATF6 pathway: As a transmembrane transcription factor, ATF6 is transported to the Golgi apparatus under ER stress conditions. There, it is cleaved by S1P and S2P proteases, releasing its cytoplasmic domain. This active fragment translocates to the nucleus and enhances the expression of ER chaperones such as glucose-regulated protein 78 in an attempt to restore protein folding capacity. Under sustained stress, these initially adaptive responses ultimately shift toward pro-apoptotic signaling, leading to intestinal epithelial cell death and disruption of barrier function, thereby establishing and perpetuating an inflammatory state.

*Prevotella(P. intermedia)* can induce endoplasmic reticulum stress in intestinal epithelial cells, generating an inflammatory environment that fundamentally disrupts gut homeostasis. This mechanism can be delineated into the following three core steps (**Figure 2**):

1. Metabolic Imbalance and Succinate Accumulation: *P. intermedia* consumes dietary fiber, thereby altering the intestinal metabolic environment. A key process in this metabolic shift is the excessive production of succinate, an intermediate of the tricarboxylic acid cycle (31). Under dysbiotic conditions, elevated succinate concentrations in the intestinal lumen lead to its uptake by intestinal epithelial cells. Intracellularly, succinate inhibits prolyl hydroxylase, resulting in the stabilization and accumulation of hypoxia-inducible factor-1α (32). This “pseudo-hypoxic” state drives a metabolic switch, increasing energy demands and protein synthesis, which in turn imposes significant stress on the endoplasmic reticulum.

2. Activation of ERS Sensors and the Unfolded Protein Response: The increased protein folding load, coupled with succinate-induced mitochondrial dysfunction and reactive oxygen species generation, disrupts the redox balance within the endoplasmic reticulum lumen. This disturbance leads to the accumulation of misfolded or unfolded proteins, thereby activating three primary ERS pathways (33) (1): The IRE1α pathway: The transmembrane kinase/endoribonuclease IRE1α undergoes oligomerization and autophosphorylation (2). The PERK-ATF4-CHOP pathway (3). The ATF6 pathway (**Figure 2**).

3. Shift in Cellular Signaling: Initially, the unfolded protein response (UPR) (34) is aimed at restoring ER homeostasis. However, chronic activation driven by persistent *Prevotella* colonization shifts the balance from adaptation to apoptosis. The IRE1α-JNK (35) and PERK-ATF4-CHOP pathways converge (36), promoting mitochondrial outer membrane permeabilization, leading to the release of cytochrome c and the activation of caspase-9 and caspase-3. This ultimately results in intestinal epithelial cell apoptosis. The loss of epithelial cells, particularly goblet cells, impairs mucus layer production and compromises tight junctions, severely disrupting intestinal barrier function. *Prevotella*-induced ERS is not triggered by a single factor but rather by a cascade initiated by metabolic perturbations (succinate/HIF-1α), leading to proteotoxic stress, UPR activation, and ultimately epithelial cell death, thereby establishing and perpetuating inflammatory responses.

#### 2.1.4 Microbial interactions trigger and amplify inflammation

The gut microbiome is now recognized as a critical modulator of inflammatory pathogenesis. Accumulating evidence suggests that specific bacterial species may promote the initiation and progression of inflammation through various mechanisms, although much of this evidence is derived from preclinical models or correlational human studies. *Fusobacterium nucleatum* is a key oncogenic microorganism associated with the development of colorectal cancer (37). It has been shown to promote tumor immune evasion by recruiting myeloid-derived suppressor cells and utilizing its surface adhesin Fap2 to suppress the cytotoxicity of natural killer cells and T cells (38). Additionally, its FadA adhesin binds to E-cadherin, activating the Wnt/β-catenin signaling pathway to drive cell proliferation. Another major participant is genotoxic *Escherichia coli* (39), which induces direct DNA double-strand breaks and chromosomal instability through the synthesis of colibactin toxin, thereby initiating mutagenesis. Similarly, enterotoxigenic *Bacteroides fragilis* secretes *B. fragilis* toxin, which cleaves E-cadherin and leads to chronic inflammation via the IL-17/STAT3 axis, thereby fostering a pro-tumorigenic microenvironment (40). Collectively, these microorganisms have been implicated in carcinogenic processes through genotoxic damage, sustained pro-inflammatory signaling, and shaping of an immunosuppressive tumor microenvironment.

**Figure 3:**
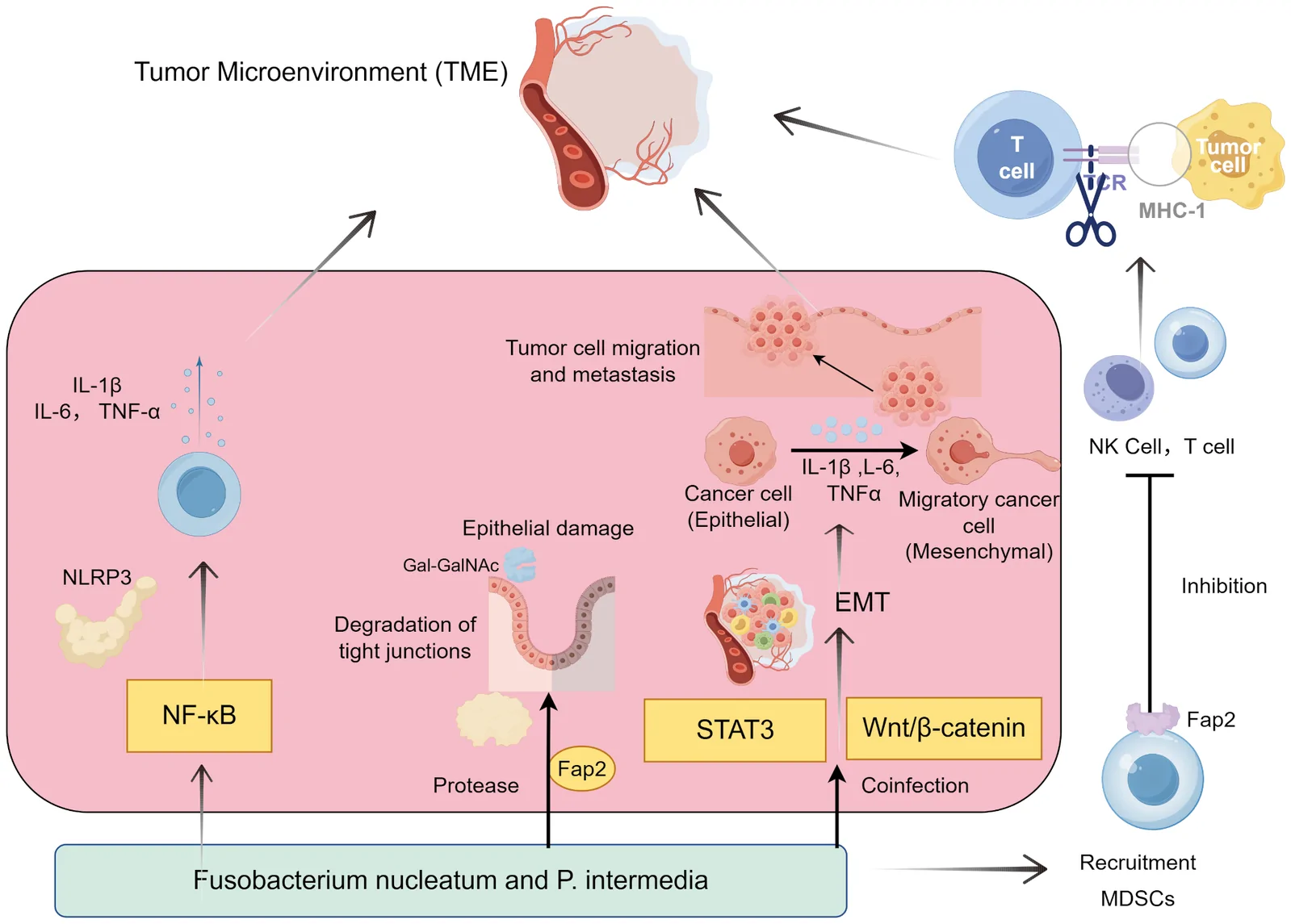
*Prevotella* and *Fusobacterium Nucleatum* form a cooperative pro-inflammatory and oncogenic network in the tumor microenvironment. *Prevotella* (exemplified by *P. intermedia*) and *Fusobacterium nucleatum* synergistically shape a pro-tumorigenic microenvironment. Firstly, they coordinately enhance inflammatory signaling by activating the NF-κB and NLRP3 inflammasome pathways, leading to excessive production of pro-inflammatory cytokines (e.g., IL-1β, IL-6, TNF-α), thereby fostering a pro-tumorigenic microenvironment (6, 111). Secondly, they synergistically disrupt epithelial integrity; *Fusobacterium nucleatum* utilizes the Fap2 adhesin to bind Gal-GalNAc on epithelial cells, while *Prevotella* secretes proteases that degrade tight junctions, thereby promoting microbial translocation and invasion. Thirdly, their co-infection synergistically activates oncogenic pathways, including Wnt/β-catenin and STAT3, promoting cell proliferation, survival, and epithelial-mesenchymal transition (EMT) (111). Notably, *P. nigrescens* has been shown to activate STAT3 signaling in esophageal squamous cell carcinoma. Additionally, they jointly modulate the tumor immune microenvironment. *Fusobacterium nucleatum* recruits myeloid-derived suppressor cells (MDSCs) and suppresses NK cell cytotoxicity, whereas *Prevotella* may enhance these effects, collectively inducing an immunosuppressive niche that permits immune evasion.

Emerging studies have highlighted the importance of microbial synergy in carcinogenesis, particularly the cooperative interactions between *Prevotella* species (e.g., *P. intermedia*, *P. nigrescens*) and *Fusobacterium nucleatum*. Both pathogens originate from the oral microbiome and can co-colonize the gastrointestinal tract and tumors, where their additive effects may be associated with exacerbated malignant progression, as suggested by *in vitro* and animal studies as well as some human observational data. The mechanisms underlying this synergy are multifaceted and may collectively induce a broader and more intense inflammatory response (**Figure 3**). However, it is important to note that most human studies reporting such synergy are correlational; although some have adjusted for potential confounders such as age, sex, and body mass index, residual confounding by dietary habits, medication use (e.g., antibiotics, proton pump inhibitors), or other lifestyle factors cannot be fully excluded.

Critically, these bacteria have been observed to co-localize with cancer cells in metastatic lesions in preclinical models, and their co-existence has been associated with enhanced chemotherapy resistance, potentially through the induction of autophagy or modulation of drug metabolism pathways. Epidemiological studies indicate that the co-enrichment of these bacteria is associated with an increased risk of colorectal, esophageal, and pancreatic cancers (41, 42), though further prospective studies with rigorous confounder control are needed to establish causality.

### 2.2 *Prevotella* promotes carcinogenesis primarily through inflammation-to-cancer transformation and other mechanisms

Having elucidated the direct mechanisms of inflammation induction, this section further explores how *Prevotella* contributes to tumorigenesis by fostering a chronic inflammatory microenvironment that shapes the tumor microenvironment (TME). This section integrates the concepts of inflammation-to-cancer transformation, bacterial toxin-induced damage, and the oral–gut axis to explain the mechanisms underlying tumor formation. But currently, most evidence is correlational; causal links in humans require validation via longitudinal or interventional studies.

#### 2.2.1 Inflammation-to-cancer transformation induced by *Prevotella*

The causal link between chronic inflammation and cancer development has been well established (41). Through epigenetic regulation, disruption of tissue structural integrity, and remodeling of the immune microenvironment,g certain pro-inflammatory *Prevotella* species (e.g., *P. intermedia*) may contribute to a microenvironment conducive to tumor initiation and progression, although most of the mechanistic evidence derives from preclinical models or correlational human studies.

At the level of epigenetic regulation, gastrointestinal inflammation associated with specific *Prevotella* species has been shown to potentially drive specific gene mutations and silence tumor suppressor genes through mechanisms such as DNA methylation, thereby promoting carcinogenesis.1.DNA methylation-mediated silencing: Pro-inflammatory *Prevotella* strains (e.g., *P. copri*, *P. intermedia*) induce chronic inflammation via TLR/NF-κB activation, leading to elevated levels of pro-inflammatory cytokines (e.g., IL-6, IL-1β). This inflammatory environment upregulates DNA methyltransferases (DNMTs) (6), resulting in hypermethylation and silencing of tumor suppressor genes such as *CDKN2A* (p16) in gastric and colorectal epithelial cells, as documented in studies on esophageal and gastrointestinal cancers (mostly observational or *in vitro*). 2.m6A RNA methylation: *Prevotella* species have been associated with upregulation of the m6A methyltransferase METTL3 in host cells, enhancing m6A modification of oncogenic transcripts such as *c-Myc* and *STAT3* (43). This modification stabilizes these transcripts via reader proteins (e.g., IGF2BP2/3), thereby potentially amplifying proliferative and anti-apoptotic signals. Concurrently, METTL3-mediated m6A methylation of *PD-L1* mRNA increases its stability, promoting immune checkpoint expression and facilitating tumor immune evasion (44). However, direct causal evidence linking *Prevotella* to these m6A changes in humans is still limited.3.Histone modification: Some *Prevotella*-derived metabolites (e.g., butyrate, which is primarily produced by anti-tumorigenic strains such as *P. melaninogenica*) can act as histone deacetylase (HDAC) inhibitors, altering chromatin accessibility and, under certain inflammatory contexts, potentially activating pro-inflammatory gene networks (45). This highlights the context-dependent nature of *Prevotella* metabolites. Through these epigenetic mechanisms, chronic inflammation may contribute to malignant transformation, though direct causal chains from specific *Prevotella* strains to human cancer epigenetics remain to be fully established.

At the level of tissue structural disruption, studies have demonstrated that inflammation induced by *P. intermedia* significantly reduces the expression levels of intestinal epithelial tight junction proteins (claudin-2, occludin, and ZO-1), thereby compromising epithelial barrier integrity in animal models and *in vitro* systems (46). This loss of barrier function not only increases intestinal permeability but also provides a route for the transepithelial translocation of bacteria and their toxins. Furthermore, certain *Prevotella* species (including *P. intermedia*) employ a unique type IX secretion system (T9SS) (47) to load a range of virulence factors onto the outer membrane surface and release them via outer membrane vesicles (OMVs). These vesicles can penetrate host tissues, potentially establishing a positive feedback loop of chronic inflammatory destruction, thereby promoting carcinogenesis through mechanisms such as loss of mucosal function and gastric metaplasia. It should be noted, however, that most T9SS-related findings originate from *in vitro* studies, and their direct role in human gastrointestinal carcinogenesis requires further investigation.

At the level of immune microenvironment remodeling, studies have shown that LPS from *P. intermedia* significantly induces macrophages to produce pro-inflammatory mediators such as inducible nitric oxide synthase (iNOS)-derived nitric oxide (NO), IL-1β, and IL-6. These inflammatory mediators promote macrophage polarization toward the pro-tumorigenic M2 phenotype (48). Concurrently, recent research has revealed that *P. timonensis* significantly enhances cell clustering between dendritic cells and CD4+ T cells (49) and promotes DC-mediated CD4+ T cell proliferation. However, this enhanced DC-T cell clustering may lead to sustained, non-protective T cell activation, ultimately resulting in T cell exhaustion. T cell exhaustion is a critical mechanism underlying tumor immune evasion, which reduces the level of immune surveillance and facilitates carcinogenesis.

#### 2.2.2 Pro-carcinogenic bacterial toxins produced by *Prevotella*

Certain *Prevotella* species, such as *P. intermedia* and *P. nigrescens*, have been shown to produce a variety of bacterial toxins, including hemolysins, endotoxins, and exotoxins (50). These toxins serve as key virulence factors and play important roles in bacterial carcinogenesis. The hemolysin produced by *P. intermedia* lyses red blood cells, facilitating bacterial acquisition of heme and iron ions and thereby enhancing its pathogenicity. This species also produces potent exotoxins; animal studies have demonstrated that these toxins induce local tissue damage, such as skin necrosis (50). Such tissue damage can lead to mucosal epithelial metaplasia in the digestive tract, thereby increasing the potential for cancer development.

#### 2.2.3 The oral–gut axis as a cross-organ driver of tumorigenesis via microbial translocation

**Figure 4:**
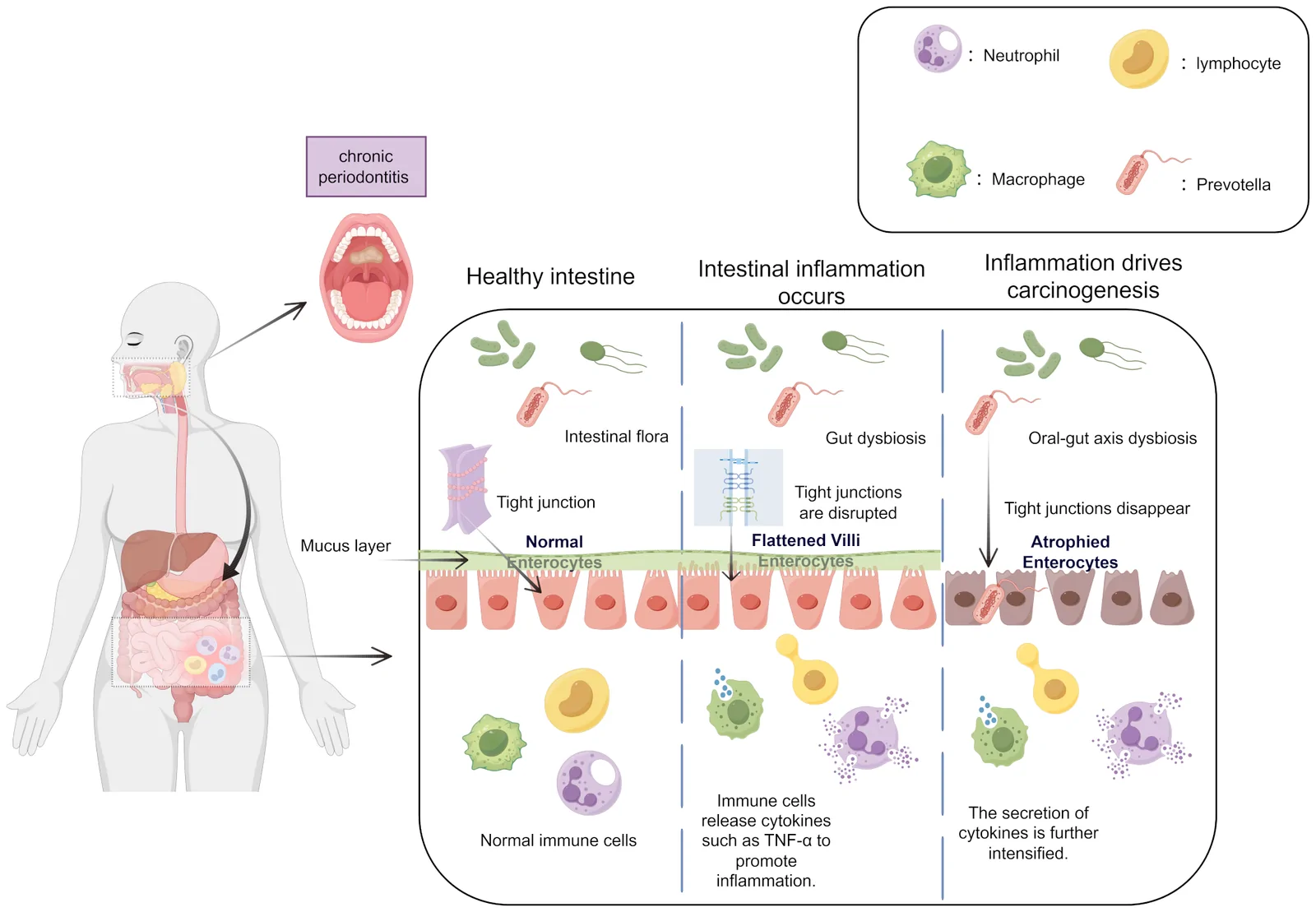
The oral–gut axis as a trans-organ driver of *Prevotella*-mediated tumorigenesis. Oral-derived microorganisms, represented by *Prevotella*, can traverse anatomical barriers and systematically influence the distal gastrointestinal microenvironment. This process is primarily accomplished through two routes: hematogenous dissemination (via the vasculature) and direct translocation (via the digestive tract). Within the oral niche, *Prevotella* constitutes a dominant microbial population alongside streptococci and *Neisseria* species. Upon successful dissemination and colonization of the gastrointestinal tract, they transition from “oral commensals” to “gastrointestinal perturbators.” The presence of *Prevotella* within the GI microbiota signifies successful cross-niche colonization. This mechanism establishes a critical pathophysiological link between oral dysbiosis and the risk of upper gastrointestinal and colorectal cancers, providing a key pathophysiological model for understanding the trans-organ origins of tumors (53).

The oral–gut axis represents an emerging research frontier in oncology (51), elucidating how dysbiosis of the oral microbiota—particularly involving *Prevotella* species such as *P. intermedia* and *P. nigrescens*—systemically influences the development of gastrointestinal (GI) tumors through microbial translocation, immune modulation, and metabolic reprogramming (52). As key pathobionts, *Prevotella* exploits host vulnerabilities (e.g., epithelial barrier defects induced by periodontitis or dietary factors) to disseminate from oral niches to the gastrointestinal tract via hematogenous or enteric routes (53) (**Figure 4**).

Following colonization, *Prevotella* promotes the formation of a tumor-permissive microenvironment primarily through the induction of immune evasion and chronic inflammation, microbial synergy, and epigenetic regulation, thereby facilitating tumorigenesis.

#### 2.2.4 Emerging evidence from recent studies

Beyond the well-characterized molecular pathways discussed above, a growing body of recent studies has provided direct evidence linking specific *Prevotella* species to cancer development across multiple gastrointestinal sites (42).

In colorectal cancer, Lo et al. (2022) (42) provided the first demonstration that *P. intermedia* is significantly enriched in colorectal adenocarcinoma tissues. Notably, co-infection with *P. intermedia* and *F. nucleatum* exerted additive effects on promoting CRC cell migration and invasion, suggesting that the co-occurrence of these oral-derived pathogens may synergistically drive malignant transformation of colorectal adenomas. A subsequent large-scale study employing rigorous confounder control (including gut transit time, fecal calprotectin, and BMI) confirmed that the association of *P. intermedia* with CRC remained robust.

In oral squamous cell carcinoma (OSCC) (54), patient-control studies have demonstrated that *P. intermedia* is detected significantly more frequently in cancer patients than in controls. Mechanistically, heat-killed *P. intermedia* promotes SCC7 cell proliferation, invasion, and proinflammatory cytokine secretion. More recently, intratumoral *P. intermedia* infection was found to aggravate OSCC progression through upregulation of ISG15 (55), which mediates M2 macrophage polarization and facilitates the formation of an immunosuppressive tumor microenvironment (56). In gastric cancer, tumor-derived *P. intermedia* has been shown to aggravate disease progression by enhancing Perilipin 3 (PLIN3) expression (57).

In esophageal squamous cell carcinoma (ESCC), *P. nigrescens*—another oral-associated *Prevotella* species—has been isolated from the gingival crevicular fluid of patients with chronic periodontitis and shown to induce upregulation of Ki67 and activation of p-STAT3 upon infection of ESCC cells (58).

In colitis-associated colorectal cancer (CAC), a *P. copri*-defined microbiota was found to mediate the oncogenic role of Zfp90 through the TLR4-PI3K-AKT-NF-κB pathway in a mouse model (59). Furthermore, a pan-cancer analysis across seven cancer types revealed that *Prevotella* is among the microbial communities shared across multiple cancers, suggesting its potential broad involvement in carcinogenesis (60).

Collectively, these recent findings—derived from human correlational studies, animal models, and *in vitro* experiments—provide growing evidence that specific *Prevotella* species are not merely passive colonizers but actively contribute to malignant progression through diverse molecular mechanisms across multiple cancer types.

## 3 Prevotella exerts antitumor and protective effects primarily through its metabolites

In contrast to its pro-tumorigenic mechanisms, a substantial body of research has revealed that certain *Prevotella* species and strains can inhibit tumor development under specific conditions (**Table 1**), primarily through the secretion of protective metabolites. However, it is important to note that most of this evidence comes from *in vitro* studies, animal models, or correlational human observations, and causal links in humans remain to be established.

### 3.1 Core antitumor mechanisms of the protective metabolite short-chain fatty acids

Although the genus *Prevotella* is often recognized for the involvement of certain pro-inflammatory species (e.g., *P. intermedia*) in tumorigenesis, multiple other species within this genus are efficient producers of short-chain fatty acids (SCFAs) (20, 61). Based on current literature, the following *Prevotella* species and strains have been identified as SCFA producers (**Table 2**).

**Table 2:** SCFA-producing *Prevotella* species and strains.

Species/strain	Key SCFA(s) produced	Evidence context	Reference(s)
*P. melaninogenica*	Acetate, propionate, butyrate	Human gut isolates; *in vitro* fermentation	(20)
*P. rothiae*	Acetate, propionate, butyrate	Human gut isolates	(20, 61)
*P.intermedia* (certain strains)	Acetate, propionate	Human gut; fiber fermentation (xylan)	(102)
*P. ruminicola* strain 23	Propionate	Rumen studies; animal model	(103)
*P. bryantii* B14	Utilizes SCFAs for growth (not a major producer)	Model organism for SCFA studies	(104)

It is worth noting that SCFA production profiles vary by species and strain. For example, *P. ruminicola* strain 23 is a well-characterized propionate producer in ruminants, whereas *P. melaninogenica* and *P. rothiae* isolated from the human gut can generate a broader spectrum including butyrate. Additionally, some *Prevotella* species may indirectly contribute to SCFA pools by producing lactate, succinate, or acetate that serve as substrates for other butyrate-producing bacteria (e.g., *Faecalibacterium prausnitzii*).

**Figure 5:**
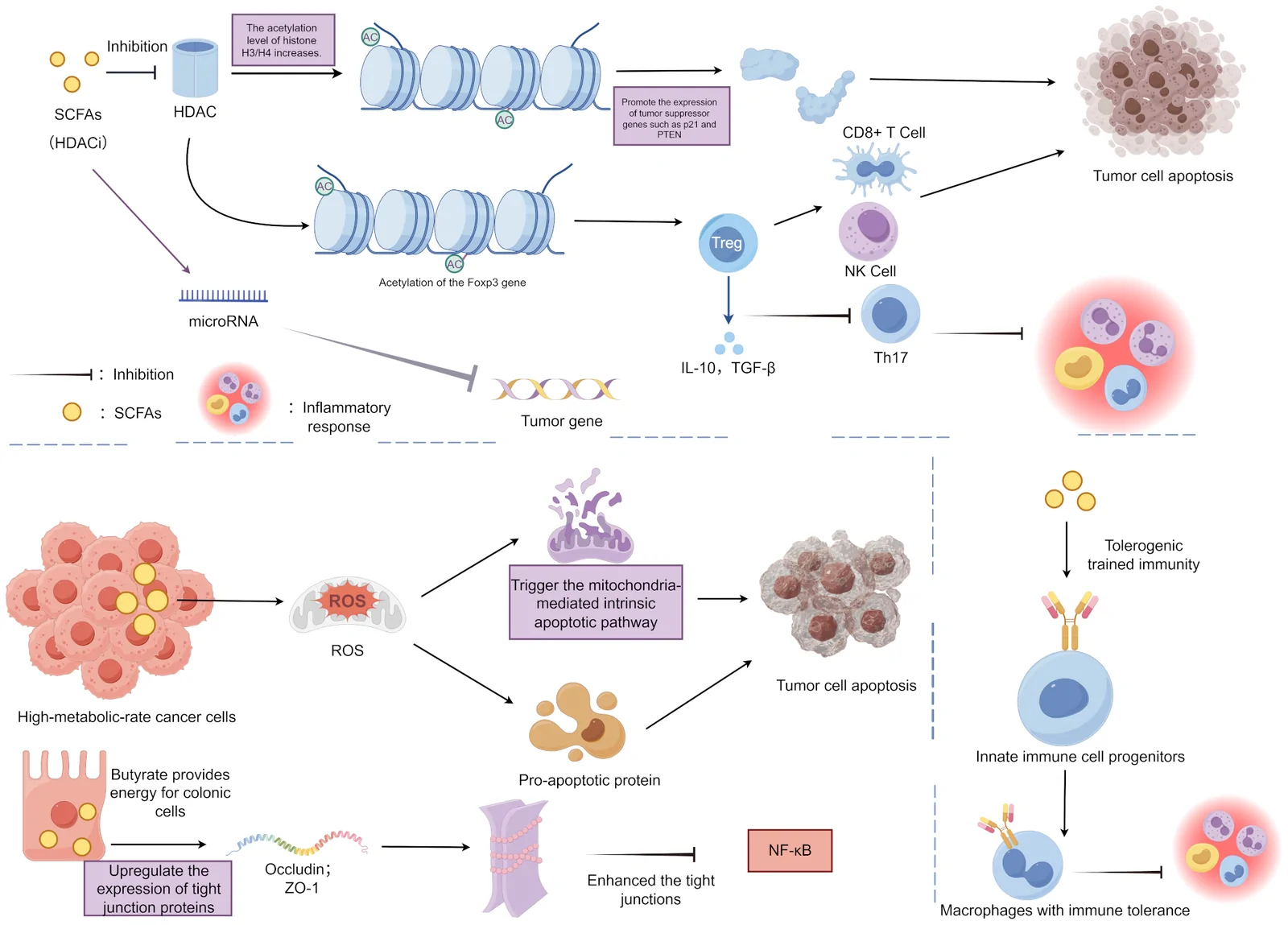
Mechanisms by which *Prevotella*-derived short-chain fatty acids induce tumor cell apoptosis through pathways. *Prevotella* ferments dietary fiber to produce butyrate, a primary metabolite that acts as a potent histone deacetylase inhibitor. Upon entering the nucleus, butyrate induces hyperacetylation of core histones, thereby opening the chromatin structure. It directly activates the transcription of tumor suppressor genes such as *p21* and *PTEN*, leading to cell cycle arrest, while also modulating microRNA expression profiles to indirectly suppress oncogene activity. Additionally, butyrate generates reactive oxygen species that act on mitochondria, triggering apoptosis in tumor cells. Furthermore, *Prevotella* reduces inflammation by enhancing intestinal epithelial tight junctions and inducing immune tolerance.

Through fermentation of dietary fiber in the gut, these SCFA-producing *Prevotella* strains primarily generate acetate, propionate, and butyrate. Emerging evidence, largely from preclinical models, indicates that these microbial metabolites—particularly butyrate—exert potent anticancer effects through multifaceted mechanisms (62), positioning certain *Prevotella* strains as potential protectors against colorectal carcinogenesis. Their antitumor mechanisms are primarily mediated through the following pathways (**Figure 5**).

#### 3.1.1 Short-chain fatty acids directly induce tumor cell cycle arrest and apoptosis and generate an antitumor environment

Butyrate, produced by certain short-chain fatty acid (SCFA)-producing *Prevotella* strains (e.g., *P. melaninogenica*, P. rothiae), has been shown to act as a potent histone deacetylase inhibitor (HDACi) in *in vitro* and animal studies (63, 64). This activity can lead to hyperacetylation of histones in the promoter regions of tumor suppressor genes such as *p21* and *PTEN* (65). Consequently, chromatin structure becomes more open, silenced gene expression may be reactivated, and cancer cell cycle arrest and apoptosis have been observed in preclinical models. Additionally, short-chain fatty acids produced by these *Prevotella* strains have been reported to promote the differentiation and function of regulatory T cells (Tregs) and antigen-presenting cells through G-protein-coupled receptor (GPCR) signaling (e.g., GPR43 (66), GPR109A) and HDAC inhibition (67). It is important to note, however, that most of these mechanistic findings are derived from preclinical models (cell lines and animal experiments), and direct human evidence linking specific *Prevotella*-derived SCFAs to these anti-tumor effects remains correlational.

In CD4+ T cells, this leads to hyperacetylation of histones at the *Foxp3* promoter (68), enhancing its transcription and promoting the differentiation and function of anti-inflammatory regulatory T cells (Tregs) (69). Furthermore, by modulating the metabolic and epigenetic states of naïve T cells, SCFAs suppress the differentiation of pro-inflammatory Th17 cells (70), thereby correcting the Th17/Treg imbalance that drives chronic inflammation. Additionally, SCFAs enhance the cytotoxic activity of CD8+ T cells and natural killer (NK) cells.

SCFAs also signal through G-protein-coupled receptors on macrophages (63) and dendritic cells to suppress their production of pro-inflammatory cytokines (e.g., IL-6, IL-12, TNF-α) in response to pathogen- or damage-associated molecular patterns (PAMPs/DAMPs), thereby modulating antigen presentation. They also promote the conversion of intestinal macrophages toward an anti-inflammatory, regulatory phenotype (63), characterized by increased IL-10 production. SCFAs and other *Prevotella*-derived metabolites can epigenetically reprogram innate immune progenitors in the bone marrow, inducing a state of “tolerance-trained immunity.” This long-term reprogramming renders macrophages less responsive to pro-inflammatory stimuli, providing a sustained anti-inflammatory tone (71). Butyrate simultaneously inhibits the recruitment and function of pro-tumorigenic immune cells such as myeloid-derived suppressor cells (MDSCs), thereby remodeling the tumor microenvironment toward an antitumor immune phenotype.

It can also suppress oncogenes by modulating microRNA expression networks. In cancer cells with high metabolic rates, butyrate is metabolized and generates excessive reactive oxygen species (ROS), triggering the mitochondria-mediated intrinsic apoptotic pathway (72). This ultimately upregulates pro-apoptotic proteins such as BAX and downregulates anti-apoptotic proteins such as BCL-2.

By maintaining immune homeostasis, *Prevotella* and its metabolites contribute to a microenvironment (73) that is less conducive to DNA damage, cell proliferation, and angiogenesis—processes fueled by chronic inflammation—thereby exerting protective effects against inflammation-driven carcinogenesis.

#### 3.1.2 *In vivo* evidence supporting the anti-tumorigenic potential of SCFA-producing *Prevotella* strains

While the HDAC inhibitory and immunomodulatory activities of butyrate have been extensively characterized *in vitro (*63, 64), recent *in vivo* evidence has substantiated the anti-tumorigenic potential of SCFA-producing *Prevotella* strains. Cogrossi et al. (2025) demonstrated that administration of the human commensal *P. melaninogenica* to transgenic Vk*MYC* mice—a model of smoldering multiple myeloma—significantly delayed disease progression. Mechanistically, *P. melaninogenica* increased SCFA production, which prevented the skewing of dendritic cells toward a pro-Th17 phenotype and subsequently reduced Th17 cell accumulation in the bone marrow. Notably, *P. melaninogenica* also uncoupled the anti-tumor efficacy from the toxicity induced by immune checkpoint blockade, suggesting its potential as an adjunctive therapeutic agent.

In human proof-of-concept studies, Bartsch et al. (2025) demonstrated that *Prevotella*-dominated individuals exhibited significantly increased fasting propionate levels upon arabinoxylan supplementation in a randomized crossover trial (74). This finding supports the concept that dietary fiber can specifically enhance the metabolic output of SCFA-producing *Prevotella* strains *in vivo*, providing a rationale for dietary interventions aimed at harnessing the protective potential of these bacteria.

#### 3.1.3 Systemic enhancement of host defense by short-chain fatty acids

SCFAs enhance intestinal barrier function (74): SCFAs, particularly butyrate, serve as the primary energy source for colonocytes and upregulate the expression of tight junction proteins such as occludin and ZO-1 (72) (**Figure 5**). This adequate energy supply reinforces tight junctions (75), reduces intestinal permeability, and prevents the translocation of luminal bacteria and their inflammatory products (e.g., LPS) into the systemic circulation (76). Consequently, this strengthens the mucosal barrier and protects against systemic endotoxemia and subsequent TLR4/NF-κB-driven inflammation (77).

## 4 Factors influencing the net effect of *Prevotella* on cancer

As discussed above, *Prevotella* exerts both pro-tumorigenic and anti-tumorigenic effects. The net outcome on carcinogenesis *in vivo* is deeply dependent on strain variability, niche context, immune microenvironment, and the overall ecological background of the host (**Table 3**).

**Table 3:** Summary of core factors influencing the functional duality of *Prevotella*.

Core determinants	Specific mechanisms	Impact on *Prevotella* function	Net effect	Reference(s)
Strain-level heterogeneity	Strain-specific genetic repertoires and metabolic preferences	Determines core metabolic outputs: SCFA production vs. inflammatory mediator generation	Protective commensal vs. Pathogenic driver	(105, 106)
Dietary patterns	Diet as primary environmental driver: High-fiber diet provides fermentable substrates; Western-style diet	Selects and directs Prevotella metabolic pathways toward SCFA production or pro-inflammatory reprogramming	Antitumor vs. Pro-tumorigenic	(74, 107)
Microbial community context	Interactions with other microbes: Co-aggregation with F. nucleatum vs. association with F. prausnitzii	Mediates synergistic pro-inflammatory effects or contributes to protective ecological networks	Enhanced pathogenicity vs. Reinforced antitumor effects	(42, 108)
Anatomical location	Tissue-specific microenvironments: Oral (periodontal niche), Gut (complex chemical/immune gradients), Vagina	Site-dependent functional outcomes: Periodontitis-associated pathobiont, context-dependent gut effects (109)	Local pro-tumorigenic effects, Context-dependent outcomes, Gynecological malignancy risk	(110)

### 4.1 Strain heterogeneity and functional divergence underlie differential functions

Genomic and functional diversity among *Prevotella* species and strains underpins their opposing roles in cancer. For instance, *P. melaninogenica* and *P. rothiae* strains are proficient in fermenting dietary fiber, yielding high levels of short-chain fatty acids (SCFAs) (20), such as butyrate. These SCFAs exert potent anti-inflammatory and anti-tumor effects through mechanisms including histone deacetylase (HDAC) inhibition, enhancement of epithelial barrier integrity, and promotion of regulatory T cell (Treg) differentiation (67). Conversely, other strains commonly enriched in dysbiotic microbiomes, such as *P. intermedia*, harbor distinct genetic repertoires. These strains may preferentially utilize mucins or dietary proteins, leading to the production of potentially deleterious metabolites and activation of pro-inflammatory pathways (e.g., IL-1β, IL-6) via toll-like receptors (TLRs) (22), thereby fostering a pro-tumorigenic microenvironment through mechanisms such as inflammation-to-cancer transformation.

### 4.2 Niche context and host diet contribute to functional divergence

The niche context of bacterial strains is a major determinant of their functional outcomes. When strains that preferentially utilize dietary fiber and produce short-chain fatty acids (SCFAs) occupy the dominant ecological niche in the gastrointestinal tract, they can shape an antitumor microenvironment. Conversely, when environmental conditions favor the dominance of strains that utilize mucins and proteins, this shift can promote inflammation and foster a tumor-supporting immune microenvironment.

Diet, as a primary shaper of the ecological niche, represents a critical factor in modulating *Prevotella* function. A high-fiber diet promotes an ecosystem dominated by fiber-utilizing, SCFA-producing strains, directing these bacteria toward SCFA production and thereby contributing to improved intestinal barrier function, systemic anti-inflammatory effects, and immune homeostasis, all of which suppress tumorigenesis. In stark contrast, a Western diet characterized by high fat and animal protein content may select for *Prevotella* strains that favor the production of inflammatory mediators (78) and disrupt intestinal barrier integrity, or alternatively, may induce a metabolic shift in existing strains. This can result in systemic endotoxemia (e.g., elevated LPS) and chronic activation of innate immune pathways (e.g., TLR4/NF-κB), creating a permissive environment for tumor initiation and progression (79).

### 4.3 Microbial community interactions and ecological context exert synergistic effects

The role of *Prevotella* is profoundly shaped by its interactions with other members of the broader microbial community, although most evidence is derived from correlational human studies or animal models. When certain pro-inflammatory *Prevotella* species (e.g., *P. intermedia*) co-occur with established pathobionts such as *Fusobacterium nucleatum* (41, 42) or other pro-inflammatory bacteria, their co-abundance has been associated with synergistic pro-tumorigenic effects in observational studies. Such collaboration may amplify inflammatory signaling, compromise host immune surveillance, and potentially enhance the stability and resilience of a dysbiotic, tumor-associated microbiome. However, it is important to note that most human studies reporting such synergy are correlational; although some have adjusted for potential confounders such as age, sex, and body mass index, residual confounding by dietary habits, medication use (e.g., antibiotics, proton pump inhibitors), or other lifestyle factors cannot be fully excluded. In contrast, when situated within a balanced microbial community dominated by SCFA-producing bacteria such as *Faecalibacterium prausnitzii* (80), certain *Prevotella* strains (particularly SCFA-producing strains like *P. melaninogenica*) are more likely to contribute to a protective, anti-tumor ecological network, though direct causal evidence remains limited.

### 4.4 Anatomical site and tissue-specific microenvironment determine pathological outcomes

The impact of *Prevotella* is highly sensitive to its anatomical location. Different sites of colonization exert distinct effects on host pathophysiology, though most evidence is derived from observational or preclinical studies.

In the oral cavity, certain *Prevotella* species (e.g., *P. intermedia*) are associated with periodontal disease (81) and have been associated with an increased risk of oral and gastrointestinal cancers in epidemiological studies, potentially by promoting local inflammation and tissue damage. However, direct causal evidence linking oral *Prevotella* to cancer risk remains limited.

In gastric cancer, *Prevotella* strains such as *P. intermedia* have been observed to frequently co-colonize with *Helicobacter pylori* (82). By activating pattern recognition receptors such as TLR2 and TLR4, they may enhance the production of pro-inflammatory cytokines (e.g., IL-1β, TNF-α) and reactive nitrogen species (RNS) (83), thereby potentially exacerbating *H. pylori*-induced chronic gastritis. Most of these mechanistic insights are derived from *in vitro* or animal models; human studies are largely correlational.

In colorectal cancer (CRC), the role of *Prevotella* is context-dependent. On one hand, specific pro-tumorigenic species such as *P. intermedia* have been associated with pro-tumorigenic phenotypes. They commonly co-aggregate with established oncogenic pathobionts like *Fusobacterium nucleatum* (42, 84), and their co-abundance has been associated with amplified pro-inflammatory signals and a tumor-permissive immune environment characterized by myeloid-derived suppressor cell (MDSC) infiltration and Th17 skewing in observational studies. However, it is important to note that while some studies have adjusted for confounders such as age, sex, and BMI, residual confounding by diet, medication, or lifestyle factors cannot be fully excluded. On the other hand, a *Prevotella*-dominated enterotype associated with high-fiber diets has been linked to better prognosis in some cohorts. Thus, the net effect in CRC is defined not by the mere presence of the genus, but by the functional output of specific strains and the prevailing metabolic and immune context within the tumor microenvironment.

For esophageal and pancreatic cancers, direct evidence remains limited yet suggestive (85). According to the oral–gut axis hypothesis (86), oral *Prevotella* species may translocate to the esophagus and pancreas (via duodenal reflux) through repeated swallowing, where they could initiate or perpetuate local inflammation and carcinogenic processes. This area represents a critical frontier for future research on microbiome-mediated carcinogenesis, but currently most evidence is correlational and requires prospective validation.

To provide a comprehensive overview of species-specific evidence, we summarize the currently reported pro-tumorigenic and anti-tumorigenic *Prevotella* species, their key mechanisms, associated cancer types, evidence levels, and corresponding references in (**Table 4**). This table highlights that the functional outcome of *Prevotella* in gastrointestinal tumorigenesis is highly species- and strain-dependent, with *P. intermedia* and *P. copri* predominantly associated with pro-tumorigenic effects, while *P. melaninogenica* and *P. rothiae* exhibit anti-tumorigenic potential primarily through SCFA production.

**Table 4:** Species-specific evidence of *Prevotella* in gastrointestinal tumorigenesis.

*Prevotella* species	Role in cancer	Key mechanisms	Cancer type(s)	Evidence level	Reference(s)
*P. intermedia*	Pro-tumorigenic	Enriched in CRC tissues; additive effects with *F. nucleatum* on CRC cell migration/invasion	CRC, gastric cancer, OSCC, HCC	Human correlational + Animal model + *In vitro*	(13) (27) (42)
*P. copri*	Pro-tumorigenic	The oncogenic effect of Zfp90 is mediated via the TLR4-PI3K-AKT-NF-κB signaling pathway in a CAC mouse model.	CRC, CAC, HCC (potential)	Human correlational + Animal model	(42) (22)
*P. nigrescens*	Pro-tumorigenic	It produces hemolysin, endotoxin and exotoxin, and is positively correlated with pancreatic cancer risk.	ESCC, pancreatic cancer	Human correlational + *In vitro*	(50)
P. timonensis	Pro-tumorigenic	Enhances the aggregation of DCs and CD4+ T cells and promotes T cell proliferation	GI cancers (speculative)	*In vitro*	(49)
*P. melaninogenica*	Anti-tumorigenic	Exerts anti-inflammatory effects via butyrate-mediated HDAC inhibition	Multiple myeloma, CRC (potential)	Animal model	(20, 61)
*P. rothiae*	Anti-tumorigenic	Ferments dietary fiber to produce SCFAs	CRC	Human isolates + *In vitro*	(20, 61)
*P. ruminicola* strain 23	Anti-tumorigenic	Efficiently produces propionate through a vitamin B12-dependent pathway	CRC	Animal model	(103)

## 5 Clinical significance and translational prospects of *Prevotella* in gastrointestinal tumors

### 5.1 Applications of microbial biomarkers in oncology

The duality of *Prevotella* in cancer pathogenesis carries potential clinical implications and opens new avenues for translational medicine. The most significant application at present lies in the discovery of microbiome-based biomarkers. The abundance of specific *Prevotella* species (e.g., *P. intermedia*), their co-aggregation with established pathobionts such as *Fusobacterium nucleatum*, and the resultant microbial synergy have been associated with prognostic value in observational studies. Detection of these features in fecal, tissue, or even blood samples may, pending prospective validation, enable refined risk stratification and prediction of tumor progression, treatment response, and overall survival in cancers such as colorectal cancer (CRC) and gastric cancer. For instance, a *Prevotella*-enriched enterotype associated with high short-chain fatty acid (SCFA) production has been linked to better prognosis and potentially increased sensitivity to immunotherapy in some cohorts, whereas a pro-inflammatory *Prevotella* signature may suggest the need for more aggressive treatment strategies.

Beyond their standalone diagnostic potential, microbiome-based biomarkers—particularly those involving specific *Prevotella* species—could be integrated with existing cancer screening modalities to enhance early detection and risk stratification.

First, microbiome signatures can complement fecal immunochemical tests (FIT) (87), the most widely used non-invasive screening tool for colorectal cancer (CRC). While FIT effectively detects occult blood, its sensitivity for advanced adenomas remains suboptimal (approximately 23–46%) (88), and it generates substantial false-positive results. Recent population-based studies have demonstrated that combining microbial profiles with quantitative FIT values improves the detection of premalignant lesions beyond optimizing FIT alone. In a Norwegian cohort of 1,034 FIT-positive participants, shotgun metagenomic profiling of FIT leftovers identified microbial signatures that helped differentiate individuals with true neoplastic findings from those who tested FIT positive for other reasons (88). This approach could reduce unnecessary colonoscopies while maintaining or improving detection sensitivity.

Second, gut microbiome analysis can enhance risk stratification for colonoscopy screening (89). Integrating lifestyle parameters with gut microbial signatures has been shown to provide effective non-invasive strategies for colorectal adenoma risk stratification. Models incorporating bacterial biomarkers such as *Fusobacterium nucleatum* and *pks*^+^ *Escherichia coli* have demonstrated robust discrimination (c-statistic = 0.84–0.86) for adenoma detection. Extending such approaches to include *Prevotella*-related signatures could further refine risk assessment, enabling personalized screening intervals—for example, earlier or more frequent colonoscopy for individuals with high-risk microbial profiles, and less frequent surveillance for those with low-risk profiles (89).

Third, the integration of microbiome data with dietary and lifestyle information offers a complementary axis for screening (90). A recent study demonstrated that combining gut microbiota data with dietary habits improved the area under the curve from 0.790 (microbiota alone) to 0.804 for distinguishing individuals with CRC-related lesions from those without pathological findings. This suggests that the interaction between gut microbiota and dietary habits is relevant when a microbial signature is used as a CRC marker.

Fourth, emerging multi-omic stool screening paradigms are integrating host epigenetic markers (e.g., DNA methylation) with gut microbiome features using artificial intelligence (91). These approaches capture complementary layers of early tumor biology—epithelial shedding reflected in host methylation signals, together with luminal ecological and inflammatory changes represented by microbial features. While *Prevotella*-specific signatures have not yet been incorporated into such multi-omic platforms, their strain-level functional heterogeneity and context-dependent roles make them promising candidates for future integration.

Finally, a potential stepwise screening strategy could be envisioned: initial non-invasive fecal microbiome screening to identify high-risk individuals based on *Prevotella*-related signatures, followed by confirmatory FIT or colonoscopy for those with high-risk profiles (91). Such an approach could improve early detection rates, reduce unnecessary invasive procedures, and enable personalized screening intervals based on individual microbial risk profiles. However, prospective validation in large screening cohorts and standardization of microbiome profiling methods remain essential prerequisites before clinical implementation.

### 5.2 Application of precise regulation of microbiome in oncology

Research into the pathogenic mechanisms of *Prevotella* has simultaneously led to a second major frontier: precision microbiome modulation. Understanding the context-dependent role of specific *Prevotella* strains facilitates the development of targeted interventions. For patients potentially harboring harmful strains, several strategies are under exploration, each with distinct feasibility profiles, risks, and translational pathways:

(1) Next-generation probiotics

One potential strategy is the development of next-generation probiotics utilizing beneficial *Prevotella* strains (e.g., SCFA-producing isolates) or other well-defined SCFA-producing bacterial species to competitively exclude pro-tumorigenic species. However, this approach carries several risks, including strain instability within the gut microbiota, unexpected pro-inflammatory effects that may depend on host diet and immune status, and difficulty achieving long-term engraftment. Therefore, the translational pathway requires rigorous strain safety profiling under GMP conditions, first-in-human safety trials, and biomarker-guided patient selection (e.g., based on baseline *Prevotella* enterotype) before any clinical application can be considered.

(2) Prebiotic and dietary interventions

A second strategy involves personalized high-fiber diets designed to direct the metabolic activity of resident *Prevotella* communities toward protective SCFA production. Key risks include high inter-individual variability in response (influenced by baseline microbiota composition, host genetics, and dietary adherence), potential gastrointestinal side effects (e.g., bloating, flatulence), and the possibility of inadvertently promoting pro-inflammatory strains if the fiber type is not matched to specific strain capabilities. To move this strategy forward, randomized controlled trials in cancer populations with repeated microbiome and metabolite monitoring are needed, along with the development of personalized fiber formulas tailored to each patient’s baseline *Prevotella* strain composition.

(3) Selective antimicrobial agents

A third approach is the use of selective antimicrobial agents, such as narrow-spectrum antibiotics, bacteriophages, or CRISPR-based agents, that specifically target pro-inflammatory *Prevotella* or *Fusobacterium nucleatum* while minimizing collateral ecological damage. The main risks include off-target effects on beneficial *Prevotella* strains or other commensals, the potential for resistance development, and microbiome dysbiosis that could lead to secondary infections (e.g., *Clostridioides difficile*). Consequently, the translational pathway demands rigorous target specificity validation, safety studies in gnotobiotic animal models, and phased clinical trials with comprehensive microbiome endpoint monitoring, all of which remain at an early preclinical stage.

Importantly, none of these strategies is ready for clinical implementation; they represent research directions that require systematic safety and efficacy evaluation before any clinical recommendation can be made.

### 5.3 Applications of multi-omics technologies in oncology

Future research must pivot from correlation to causation and from genus-level to strain-level investigation. The emerging multi-omics technologies provide powerful tools to achieve this transition.

Metagenomic sequencing with strain-level resolution can distinguish functional differences among *Prevotella* strains that are obscured by 16S rRNA-based genus-level analyses (14, 60). For instance, Fehlner-Peach et al. demonstrated that different *P. copri* isolates possess distinct polysaccharide utilization loci, correlating with their metabolic capabilities (92).

Metatranscriptomics can reveal the real-time transcriptional activity of *Prevotella* within the tumor microenvironment, distinguishing metabolically active strains from passive colonizers and identifying which metabolic pathways (e.g., SCFA synthesis vs. virulence factor expression) are actively engaged in tumor tissues.

Metabolomics enables direct quantification of SCFAs and other microbial metabolites, establishing functional links between specific *Prevotella* strains and their metabolic outputs. Hong et al. integrated 16S rRNA sequencing with untargeted metabolomics in a mouse model of inflammation-induced colorectal tumors, identifying *Prevotella* among the key differential microbes (93). In gastric cancer patients, Liu et al. demonstrated that reduced butyrate levels distinguished cachectic from non-cachectic patients (AUC 0.792), highlighting the diagnostic potential of SCFA profiling (11, 94).

Integration of host genomics and epigenomics can elucidate how host genetic backgrounds modulate *Prevotella* colonization and function. Liang et al. demonstrated that multi-omics integration combining gut microbiota, metabolome, and host genome revealed previously unknown links between intestinal microbiota alterations and host genomics in right- versus left-sided colon cancer (95). Similar approaches could be applied to dissect how host genetic variants influence *Prevotella*-mediated inflammation and carcinogenesis.

Emerging spatial omics and single-cell technologies offer unprecedented opportunities to map the spatial distribution of *Prevotella* within the tumor microenvironment and dissect its interactions with specific immune cell subsets. These approaches could resolve whether *P. intermedia* predominantly localizes to the invasive front or necrotic cores of tumors, and which immune cell types it engages.

Integrating multi-omics data with machine learning represents a promising frontier for predictive modeling (96). Recent studies have developed multi-modal survival prediction frameworks (e.g., HMTsurv) that integrate histopathology, transcriptomics, and microbiome features to achieve superior prognostic accuracy across multiple cancer types (92). Extending such approaches to incorporate *Prevotella*-specific strain and metabolic features could enable precise risk stratification and guide personalized microbiome-based interventions (96).

Ultimately, multi-modal predictive models integrating microbiome data (strain composition), metabolomic profiles (SCFA levels), host genetic/epigenetic markers, and clinical factors will enable precise risk stratification and guide personalized microbiome-based interventions. Integrating large-scale clinical cohorts with these multi-omics technologies, combined with patient-derived organoid models, will enable systematic dissection of strain-specific functions of *Prevotella* and their interactions with host genetic backgrounds, dietary habits, and commensal microbial communities in humans.”

Finally, the role of *Prevotella* underscores the importance of integrating microbiome analysis into clinical trial design. Future oncology trials, particularly those involving immunotherapy, could incorporate longitudinal microbiome monitoring to understand how treatments alter the microbial landscape and how these changes, in turn, affect efficacy and toxicity. This “microbiome-first” approach in drug development may help unravel mechanisms of resistance and identify patients most likely to benefit from combination therapies that include microbiome modulators. In summary, transitioning from correlational to causal understanding of *Prevotella*’s role—through mechanistic studies, prospective cohorts, and interventional trials—will be essential to unlock its potential as a diagnostic tool, therapeutic target, and modulator of cancer treatment, ultimately enabling rational approaches to personalized oncology care.

## 6 Conclusion

As a core genus within the gastrointestinal microecology, different species and strains within the genus *Prevotella* exhibit a complex dual role in the development of digestive tract tumors, based largely on preclinical models and correlational human studies. On one hand, specific pro-tumorigenic strains (e.g., *P. intermedia*) have been associated with fostering a pro-tumorigenic microenvironment by activating the TLR4/NF-κB and TLR2/Th17/IL-17 inflammatory axes, synergizing with *Fusobacterium nucleatum*, inducing epigenetic reprogramming, and triggering endoplasmic reticulum stress. On the other hand, certain SCFA-producing strains (e.g., *P. melaninogenica*, *P. rothiae*) have been shown to exert anti-tumorigenic effects by fermenting dietary fiber to produce short-chain fatty acids (SCFAs), which enhance intestinal barrier function, inhibit histone deacetylases, and promote regulatory T cell differentiation. This functional duality appears to be governed by the interplay of strain heterogeneity, host dietary patterns, microbial community context, and anatomical localization.

However, clinical translation still faces critical bottlenecks. Existing evidence is largely derived from correlational studies, with insufficient causal validation. Core mechanisms are primarily based on animal models, and direct evidence of how strain-specific functions are modulated by the microenvironment in humans remains lacking. Furthermore, traditional taxonomic analyses often fail to capture functional differentiation at the strain level, impeding the development of precision intervention strategies.

Elucidating the role of the oral–gut axis in cross-organ translocation and distant carcinogenesis may provide novel targets for challenging malignancies such as esophageal and pancreatic cancers. Concurrently, developing highly sensitive predictive models based on strain functional characteristics will facilitate risk stratification and prognostic assessment.

Ultimately, these advances may lay the foundation for precision microecological interventions targeting *Prevotella*: enriching beneficial SCFA-producing strains through dietary fiber, developing next-generation probiotics, and precisely eliminating pro-tumorigenic subspecies using bacteriophages or narrow-spectrum antimicrobials to restore intestinal microecological balance and antitumor immune surveillance. Advancing the field from correlational studies toward clinical translation will offer innovative solutions for optimizing prevention strategies, enhancing therapeutic efficacy, and improving patient outcomes in gastrointestinal tumors, though many of these approaches remain at the preclinical stage.
